# Efficient generation of few-cycle pulses beyond 10 μm from an optical parametric amplifier pumped by a 1-µm laser system

**DOI:** 10.1038/s41598-022-08964-w

**Published:** 2022-03-24

**Authors:** Zsuzsanna Heiner, Valentin Petrov, Vladimir L. Panyutin, Valeriy V. Badikov, Kiyoshi Kato, Kentaro Miyata, Mark Mero

**Affiliations:** 1 School of Analytical Sciences Adlershof, Humboldt-Universität zu Berlin, 12489 Berlin, Germany; 2 Max Born Institute for Nonlinear Optics and Short Pulse Spectroscopy, 12489 Berlin, Germany; 3 High Technologies Laboratory, Kuban State University, Krasnodar, Russia 350040; 4 Chitose Institute of Science and Technology, Chitose, Hokkaido 066-8655 Japan; 5 Okamoto Optics Inc., Yokohama, Kanagawa 235-0008 Japan; 6 RIKEN Center for Advanced Photonics, RIKEN, Wako, Saitama 351-0198 Japan

**Keywords:** Ultrafast lasers, Nonlinear optics

## Abstract

Nonlinear vibrational spectroscopy profits from broadband sources emitting in the molecular fingerprint region. Yet, broadband lasers operating at wavelengths above 7 μm have been lacking, while traditional cascaded parametric frequency down-conversion schemes suffer from exceedingly low conversion efficiencies. Here we present efficient, direct frequency down-conversion of femtosecond 100-kHz, 1.03-μm pulses to the mid-infrared from 7.5 to 13.3 μm in a supercontinuum-seeded, tunable, single-stage optical parametric amplifier based on the wide-bandgap material Cd_0.65_Hg_0.35_Ga_2_S_4_. The amplifier delivers near transform-limited, few-cycle pulses with an average power > 30 mW at center wavelengths between 8.8 and 10.6 μm, at conversion efficiencies far surpassing that of optical parametric amplification followed by difference-frequency generation or intrapulse difference-frequency generation. The pulse duration at 10.6 μm is 101 fs corresponding to 2.9 optical cycles with a spectral coverage of 760–1160 cm^−1^. Cd_x_Hg_1−x_Ga_2_S_4_ is an attractive alternative to LiGaS_2_ and BaGa_4_S_7_ in small-scale, Yb-laser-pumped, few-cycle mid-infrared optical parametric amplifiers and offers a much higher nonlinear figure of merit compared to those materials. Leveraging the inherent spatial variation of composition in Cd_x_Hg_1−x_Ga_2_S_4_, an approach is proposed to give access to a significant fraction of the molecular fingerprint region using a single crystal at a fixed phase matching angle.

## Introduction

Generation and amplification of broadband, coherent mid-infrared (MIR, 3–30 µm) laser pulses at repetition rates > > 1 kHz can tremendously benefit vibrational and strong-field spectroscopy^1–3^. Of particular interest is the range between 7 and 20 µm (i.e., 500–1400 cm^−1^ or 15–43 THz), the so-called fingerprint region, where characteristic bending vibrational bands of complex molecules reside. Currently, only gas lasers emitting at discrete lines and narrowband quantum cascade lasers with limited tunability are available in the MIR, while broadband solid state lasers are extremely rare^4^ and are restricted to wavelengths below 5 µm. On the other hand, optical parametric frequency down-conversion of ultrafast near-infrared laser pulses based on three-wave interaction in nonlinear optical (NLO) crystals has been a well-established method to cover the MIR spectral range in a gapless manner^5^. Nowadays, diode-pumped ultrafast Yb laser technology represents the most mature, power-scalable laser concept for broadband frequency down-conversion. Such amplified pump sources are capable of generating supercontinua in a straightforward manner to seed optical parametric amplifiers (OPAs) or can be post-compressed to enable robust difference frequency mixing between the blue and red components within a single laser pulse to reach the MIR range in a process called intrapulse difference frequency generation (DFG).

One of the disadvantages of employing Yb lasers for frequency down-conversion to the MIR beyond 7 µm is the large quantum defect, which severely restricts the energy conversion efficiency. A further limitation is that there are only a handful of wide-bandgap NLO crystals that can be pumped by ultrafast pulses near 1 µm without nonlinear absorption and optical damage and are also transparent above 7 µm. The only commercially widely available non-oxide material with such properties is AgGaS_2_ (AGS), but its relatively low, 2.7-eV bandgap energy and low heat conductivity lead to low damage threshold when pumped at 1 µm. Therefore, the traditional approach has been to employ a cascaded frequency mixing scheme based on optical parametric amplification in a wide-gap oxide crystal followed by difference frequency mixing of the signal and idler beams in a narrow-gap non-oxide crystal. Despite using non-oxide crystals with high nonlinear figure of merit, the cascaded scheme typically suffers from low energy conversion efficiencies (i.e., few 0.1%). Approaches that can improve conversion efficiency are therefore highly sought after.

In the last few years, LiGaS_2_ (LGS) has received a lot of attention as a novel wide-bandgap NLO material for broadband MIR optical parametric converters^6,7^. LGS exhibits a bandgap energy of 4.15 eV, while its infrared absorption edge is at ~ 12 μm (ref.^8^). Thanks to the short UV cutoff wavelength of LGS, the crystal has been successfully used in both 1-µm-pumped OPAs^9–12^ and intrapulse DFG units supporting broadband operation^13,14^. More recently, BaGa_4_S_7_ (BGS) has emerged as an even more promising material for power scaling due to its availability in larger single-crystal sizes than LGS (see e.g., ref.^10^), while having similar linear and nonlinear optical properties and a longer IR cutoff wavelength compared to LGS (ref.^15–19^). BGS was also demonstrated to support few-cycle MIR pulse durations even in relatively long crystals, importantly at longer wavelengths than LGS (ref.^17^). Even though the effective nonlinearity of LGS and BGS is much lower than that of commercially widely available narrow-bandgap non-oxide crystals, such as GaSe and AgGaSe_2_ (applicable in cascaded schemes), and also AGS, the use of LGS and BGS has been shown to drastically improve the overall conversion efficiency compared to that of cascaded schemes^17^.

Mixing isomorphous non-oxide crystals may also offer improved conversion efficiency in 1-µm-pumped parametric devices. In comparison with the parent compounds, mixed crystals can feature improved thermo-mechanical properties and bandgap energies or dispersion properties that are better suited for broadband operation in the MIR region^5^. For example, the solid solution of defect chalcopyrites, CdGa_2_S_4_ (CGS) and HgGa_2_S_4_ (HGS), has been shown to exhibit a linearly adjustable bandgap energy depending on Cd content *x* in the compound Cd_x_Hg_1-x_Ga_2_S_4_ (CHGS) with a value of 2.79 eV at *x* = 0 to 3.58 eV at *x* = 1, while leaving the long-wavelength transparency limit at the few cm^−1^ absorption level at ~ 13 µm unaffected (ref.^20^). The effective nonlinearity in type-I interaction at *ϕ* = 45° is given by *d*_eff_ = *d*_36_ sin(θ), where *d*_36_(CHGS) = 25 pm/V, which is much larger than the *d*_eff_ values of LGS and BGS, and even that of AGS under identical conditions with *d*_36_(AGS) ⁓ 14 pm/V (ref.^5^). More importantly, the figure of merit of the above OPA process, [*d*_eff_(θ)]^2^/[*n*(*λ*_p_,θ) *n*(*λ*_s_) *n*(*λ*_i_)], is ~ 9 times higher for Cd_0.65_Hg_0.35_Ga_2_S_4_ used in this work than for AGS, when obtaining phase matching for an idler wavelength of 11 µm.

The main problem with such mixed crystals is the variation of the bulk composition^5^. Nevertheless, the gradient of inhomogeneity is low enough to support MIR pulse energies at sufficiently high beam quality up to the µJ level. Accordingly, OPAs based on CHGS pumped at 1250 nm by a Cr:forsterite laser^21^ and at 800 nm by a Ti:sapphire laser^22^ have been successfully demonstrated. Here we show that at limited laser spot diameters characteristic of small-scale systems, CHGS is an attractive alternative to LGS, BGS, and AGS in parametric devices pumped by ultrafast Yb lasers. In addition, we demonstrate that CHGS also allows broadband phase matching to generate MIR idler pulses in a collinear geometry, interestingly at a longer IR wavelength than LGS and BGS.

## Methods

**Figure 1:**
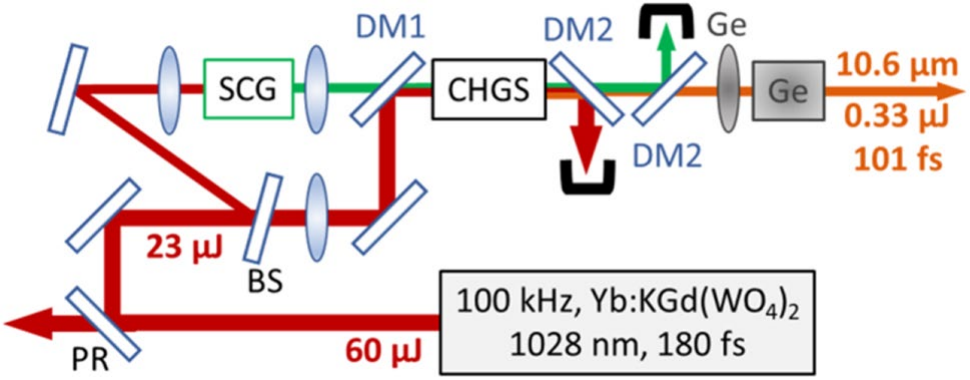
Schematic layout of the mid-infrared OPA. *PR* partial reflector, *BS* beam sampler, *SCG* supercontinuum generator based on a 6-mm-long YAG crystal, *DM1* dichroic mirror, high reflection at 1.03 µm and high transmission at > 1.1 µm, *DM2* dichroic mirror, high reflection at 1.0–1.2 µm and high transmission at 6–12 µm, *CHGS* 2-mm-long, uncoated Cd_0.65_Hg_0.35_Ga_2_S_4_ crystal, *Ge* germanium.

The schematic layout of our MIR OPA is shown in Fig. 1. The pump source is a commercial Yb:KGd(WO_4_)_2_ laser/amplifier (Pharos SP, Light Conversion Ltd.), which provides an average power of 6 W at a center wavelength of 1.028 µm with 180-fs pulse duration. A 2.3-W portion with 23 µJ/pulse is used to generate the seed pulses and to pump the MIR OPA. The remaining power is used for pumping another arm of a home-built vibrational sum-frequency generation (VSFG) spectrometer^23^. The supercontinuum seed pulses are generated by focusing < 1 μJ/pulse by an *f* = 150 mm singlet lens into a 6-mm-long, uncoated YAG crystal (cf. SCG in Fig. 1). A 19-mm achromat refocuses the supercontinuum pulses through a 1.06-μm long-pass filter and a beam combining dichroic mirror (DM1) in front of the CHGS crystal.

The spectral range 1.11–1.19 µm with positive chirp and a 1/e^2^ incident beam diameter of 710 µm is amplified in the OPA. The remaining pump beam with 22 µJ/pulse is focused by an *f* = 1000 mm lens and is combined with the seed pulses at DM1. The pump focus is also placed in front of the CHGS crystal to provide a diverging beam. The pump pulse energy incident on the OPA with a diameter of 920 µm is 19.5 µJ with a corresponding peak intensity of 32 GW/cm^2^. The OPA crystal is a 2-mm-long, uncoated Cd_0.65_Hg_0.35_Ga_2_S_4_ cut for type-I phase matching at θ = 77° and *ϕ* = 45°. The generated idler pulses pass through a pair of custom-made dichroic mirrors (DM2) with high reflectivity in the spectral range of the pump and signal beams. An *f* = 200 mm anti-reflection (AR) coated germanium (Ge) lens collimates the MIR output pulses. The residual negative group delay dispersion is removed by a bulk Ge compressor.

## Results and discussion

**Figure 2:**
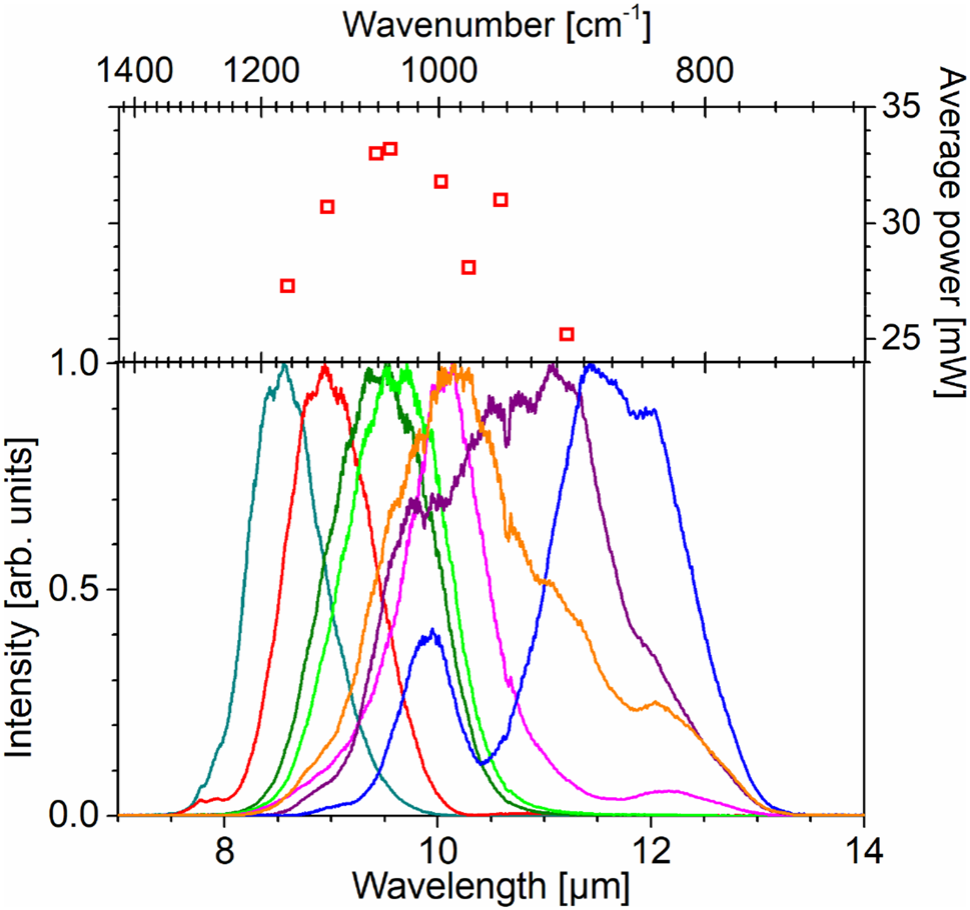
Top: Average power of the idler output beam as a function of center wavelength across the tuning range. Bottom: Representative idler spectra across the tuning range.

The average idler power measured after the germanium lens is > 30 mW in a tuning range of 8.8–10.6 µm (cf. Fig. 2, top panel). Below, unless otherwise noted, we will always refer to the power values measured after the Ge lens, which represents the practically relevant, useful output power. The overall pump-to-idler energy conversion efficiency defined as the ratio of the idler pulse energy after the germanium lens and the pump power incident on the CHGS crystal is 1.7%.

No experimental data are available on the laser-induced damage threshold of CHGS in the sub-ps pulse duration range. According to previous OPA development based on CHGS, no laser damage was observed in a 3-mm thick Cd_0.35_Hg_0.65_Ga_2_S_4_ sample up to an on-axis pump intensity of 200 GW/cm^2^ with 90-fs pulses at 820 nm and 1 kHz (ref.^22^) and in a 2-mm thick Cd_0.54_Hg_0.46_Ga_2_S_4_ crystal for pump intensities up to 170 GW/cm^2^ with 160-fs pulses at 1.25 µm, 1 kHz (ref.^21^). At our maximum pump intensity of 32 GW/cm^2^ neither laser-induced damage, nor detrimental nonlinear absorption were observed. Thanks to the exceptionally high figure of merit of the material, high enough gain can be reached at our moderate pump intensity despite the small crystal thickness.

The bottom panel of Fig. 2 shows the idler spectra obtained by sum-frequency mixing between the idler pulses and spectrally quasi-monochromatic pulses at 514 nm at a planar metal interface using a home-built VSFG spectrometer^23^. Spectral tuning is achieved by adjusting the seed-pump delay and the crystal tilt angle. The longest wavelength achieved is 13.3 µm possibly limited by the IR cutoff of CHGS. The shortest wavelength is 7.5 µm, which is determined by phase-matching.

**Figure 3:**
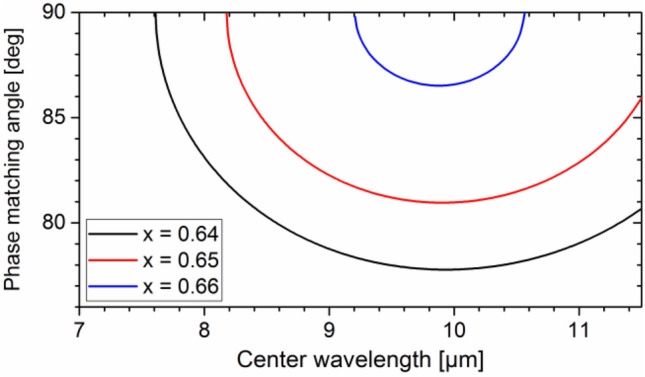
Phase matching curves for type-I OPA in Cd_x_Hg_1−x_Ga_2_S_4_ with a Cd content of *x* = 0.64 (black), 0.65 (red), and 0.66 (blue), at 20 °C and a pump wavelength of 1.028 µm.

The Sellmeier equations of Cd_x_Hg_1-x_Ga_2_S_4_ are typically obtained by linear interpolation of the squared refractive indices of HGS and CGS based on the value of *x* (ref.^24^) although direct measurements for some specific compositions also exist. However, since CGS does not exhibit sufficient birefringence for phase matching, obtaining reliable Sellmeier equations for this material has been challenging. The situation is aggravated by the fact that determining the actual value of parameter *x* is hindered by the spatial inhomogeneity of Cd_x_Hg_1-x_Ga_2_S_4_. Therefore, large deviations between the predicted and measured spectral tuning range have been observed^21,22,24^. The Sellmeier equations of CGS have been recently refined^25^. Figure 3 shows the resulting phase matching angle based on refs.^24,25^ as a function of idler wavelength for Cd_0.65_Hg_0.35_Ga_2_S_4_, when pumped at 1.028 µm (cf. red curve) in reasonable agreement with our experimental tuning range (8.5–11.2 µm) and the wavelength of broadest bandwidth (10.5 µm). We also plotted the phase matching curve for *x* = 0.64 (black curve) and 0.66 (blue curve) predicting a markedly strong effect of the composition. The plotted wavelength range is limited to 11.5 µm to conform to the range of validity of the Sellmeier equations. We note that dispersion equations predict that a Cd content of *x* ~ 0.59 will give access to wavenumbers up to the amide I vibrational band in an angle-tuning fashion down to the low-wavenumber absorption cutoff of CHGS.

**Figure 4:**
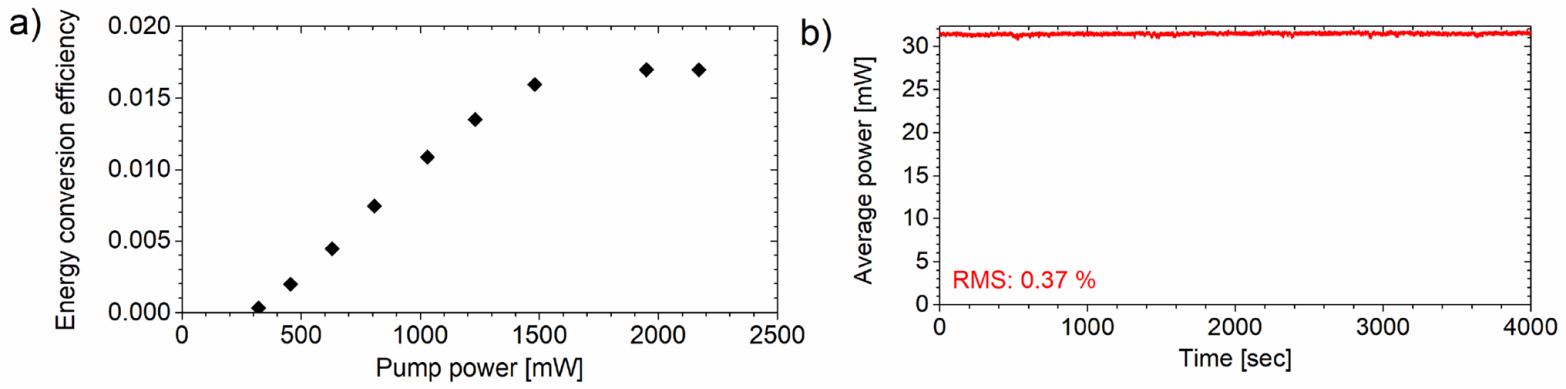
(**a**) Pump-to-idler energy conversion efficiency at 10.6 μm as a function of average pump power at 1.028 μm. The pump power was not corrected for Fresnel losses at the front face of the uncoated OPA crystal and the MIR power was measured at the output after the germanium lens. (**b**) Average power stability of the idler beam at 10.6 µm as a function of time.

The energy conversion efficiency as a function of pump power is shown in Fig. 4a confirming that saturation is achieved in the single-stage OPA already at a pump pulse energy of 19.5 µJ, which was used further on. When correcting for the losses due to Fresnel reflections at the NLO crystal, DM2 dichroic optics, and Ge lens, the energy and photon conversion efficiency is calculated to be 2.9% and 30%, respectively. Higher idler powers are feasible using better optimized coatings on transmissive optics and an AR-coated OPA crystal.

Figure 4b shows the average power stability of the idler beam measured over a period of  > 1 h on a thermal power head with a 0.6-s response time. The corresponding root-mean-square (RMS) fluctuations are at the < 0.4% level demonstrating that no laser-induced deterioration or thermal dephasing occurs in the CHGS crystal under our experimental conditions.

**Figure 5:**
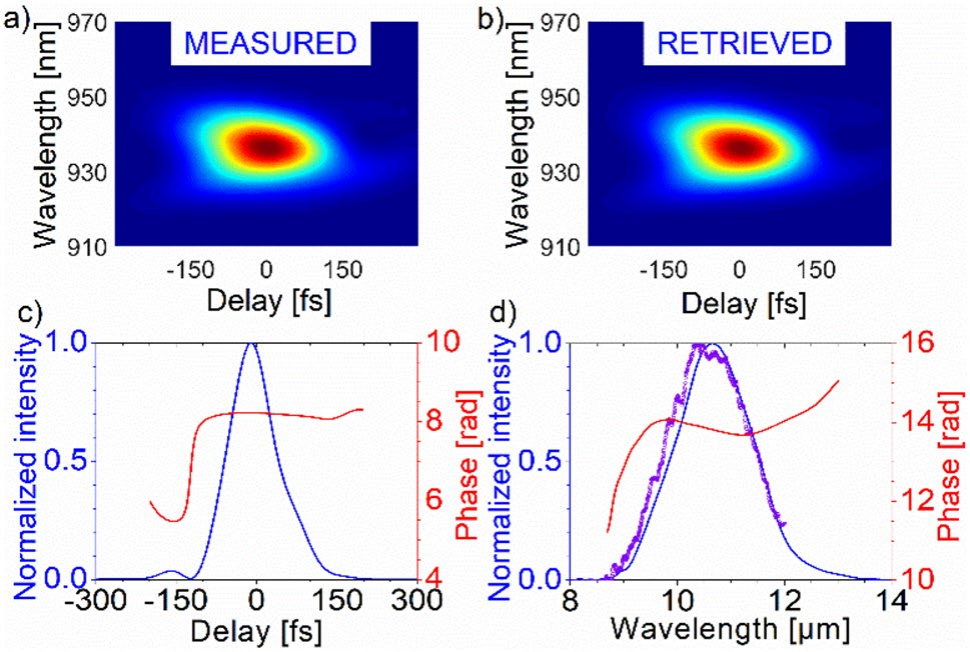
(**a**) Measured and (**b**) reconstructed X-FROG traces obtained for chirp-compensated idler pulses. Retrieved temporal (**c**) and spectral (**d**) intensity and phase. The retrieved pulse duration is 101 fs. The symbols in (**d**) represent the measured spectrum.

The negative chirp of the idler pulses is compensated by propagation through the AR-coated Ge collimating lens and AR-coated, Ge windows with a total thickness of 23 mm. The pulse duration at 10.6 µm was measured using a home-built cross-correlation frequency-resolved optical gating (X-FROG) device based on sum-frequency generation. The nonlinear element was a 200-µm-thick LGS crystal. The well-characterized pulses from the pump laser served as the gate pulses^11^. The measured and reconstructed X-FROG traces and the retrieved temporal and spectral profiles are shown in Fig. 5. The spectrum was also measured using a commercial Fourier-transform optical spectrum analyzer with an operating range of 1–12 µm, showing good agreement with the reconstructed data. The retrieved pulse duration is 101 fs corresponding to 2.9 optical cycles at 10.6 µm, which is 22% longer than the Fourier-limited value.

**Figure 6:**
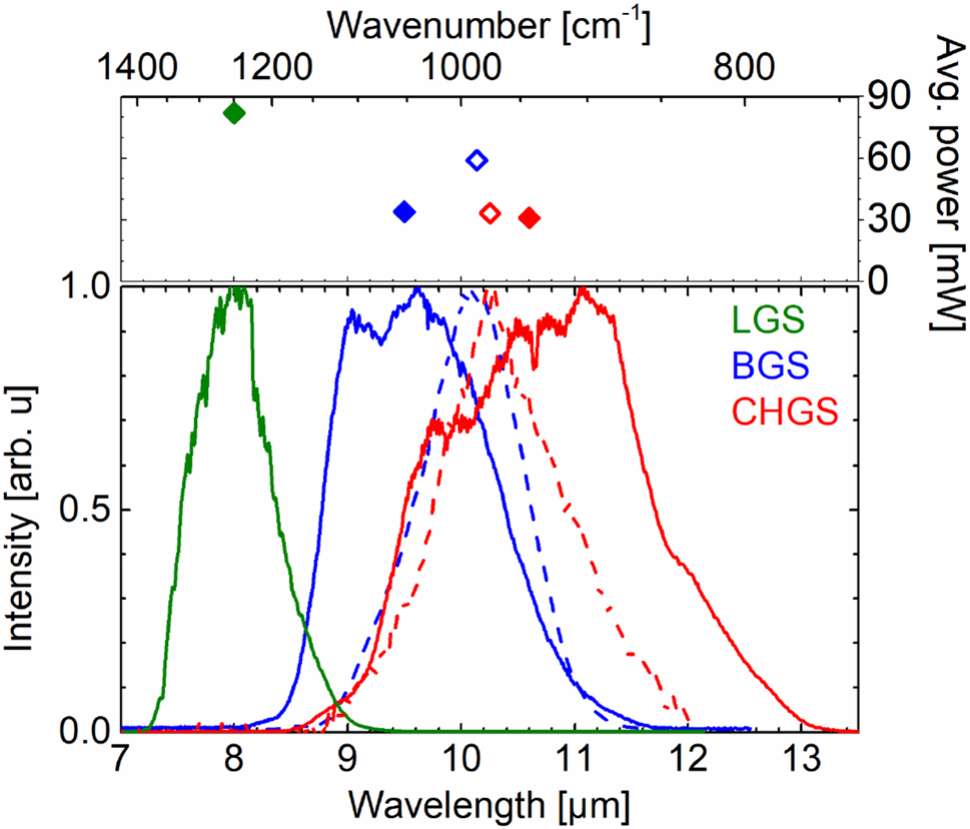
Idler spectra with broadest bandwidth (solid lines and symbols) and highest power (dashed lines and open symbols) and the corresponding average power delivered by a single-stage OPA based on an 8-mm-long LGS (ref.^17^), an 8.3-mm-long BGS (ref.^17^), and a 2-mm-long Cd_0.65_Hg_0.35_Ga_2_S_4_ (this work).

In Fig. 6, we compare the idler spectra with the corresponding power we achieved using an 8.0-mm-long LGS (ref.^17^), 8.3-mm-long BGS (ref.^17^), and a 2-mm-long CHGS crystal (this work). The single-stage OPA setup, focusing conditions, and spot sizes on crystals were identical except for the applied pump power, which was 3.4 W, 3.2 W, and 1.95 W for LGS, BGS, and CHGS, respectively. Spectra with the broadest bandwidth (solid lines) and those corresponding to the highest power (dashed lines) are shown. For LGS, the broadest spectrum corresponded to the highest power. As shown by Fig. 6, CHGS operates at quantum conversion efficiencies on par with LGS and BGS, but at longer wavelengths with broader widths than those crystals. We note that the effect of the large difference in crystal thicknesses in the three cases is diminished by the fact that the main part of the idler pulse energy is generated at the rear surface, as is common in high-gain optical parametric pre-amplifiers^22^.

## Conclusions

In summary, we employ a 2-mm-thick CHGS crystal for the generation of few-cycle MIR idler pulses using a supercontinuum-seeded single-stage OPA pumped by a 1.028-µm Yb laser. The OPA operates in the 7.5–13.3-µm spectral range, extending beyond the long-wavelength cutoff of both LGS and BGS. Thanks to the high nonlinear figure of merit of CHGS, the quantum efficiency reaches 30% at 10.6 µm. Starting from a pump power of ⁓ 2 W at a repetition rate of 100 kHz driving the entire setup, the CHGS-OPA delivers an output power of 33 mW at 10.6 µm. The overall pump-to-idler conversion efficiency exceeds considerably that of traditional cascaded frequency down-conversion schemes. Reaching higher output powers is straightforward using AR-coated crystals and better optimized coatings on dichroic optics. Chirp compensation is achieved by propagation through bulk germanium leading to 101-fs pulses (i.e., 2.9 optical cycles). Despite the low total pump pulse energy, the MIR output pulses can drive a broad range of nonlinear and/or vibrational spectroscopic applications, such as broadband vibrational sum-frequency generation spectroscopy of heterogeneous biological interfaces^26^.

We conclude that thanks to its exceptionally high nonlinearity, Cd_x_Hg_1−x_Ga_2_S_4_ is an attractive material in small-scale, ultrafast 1-µm-pumped MIR OPAs, where the use of small spot sizes can alleviate phase mismatch across the beam aperture due to the inherent composition variation of the crystal. Note that apart from making this inhomogeneity of the properties tolerable, the use of a shorter OPA crystal compared to previous work with single stage OPAs based on LGS and BGS has a few additional advantages: (1) it allows one to better utilize the upper transmission limit of a nonlinear crystal since all such OPAs operate close to this limit; (2) it enhances the parametric gain bandwidth; and (3) it suppresses higher order nonlinear effects. Finally, since the composition variation is much stronger along the growth direction, a specially designed sample of Cd_x_Hg_1−x_Ga_2_S_4_ grown along the propagation direction will support even larger bandwidths at a fixed phase-matching angle, acting similar to multiple plates cut at different angles. Note that such variation of the composition of Cd_x_Hg_1−x_Ga_2_S_4_ has been previously exploited for tuning of nanosecond optical parametric oscillators^20^.
